# Radiobrominated benzimidazole-quinoline derivatives as Platelet-derived growth factor receptor beta (PDGFRβ) imaging probes

**DOI:** 10.1038/s41598-018-28529-0

**Published:** 2018-07-10

**Authors:** Nurmaya Effendi, Kenji Mishiro, Takeshi Takarada, Akira Makino, Daisuke Yamada, Yoji Kitamura, Kazuhiro Shiba, Yasushi Kiyono, Akira Odani, Kazuma Ogawa

**Affiliations:** 1 Kanazawa University, Graduate School of Pharmaceutical Sciences, Kakuma-machi, Kanazawa, 920-1192 Japan; 2 Universitas Muslim Indonesia, Faculty of Pharmacy, Urip Sumiharjo KM. 10, Makassar, 90-231 Indonesia; 3 Kanazawa University, Institute for Frontier Science Initiative, Kakuma-machi, Kanazawa, 920-1192 Japan; 4 Okayama University, Graduate School of Medicine, 2-5-1 Shikata-cho, Okayama, 700-8558 Japan; 5 University of Fukui, Biomedical Imaging Research Center, 23-3 Matsuoka Shimoaizuki, Yoshida, 910-1193 Japan; 6 Kanazawa University, Advanced Science Research Centre, 13-1 Takara-machi, Kanazawa, 920-8640 Japan

## Abstract

Platelet-derived growth factor receptor beta (PDGFRβ) affects in numerous human cancers and has been recognized as a promising molecular target for cancer therapies. The overexpression of PDGFRβ could be a biomarker for cancer diagnosis. Radiolabeled ligands having high affinity for the molecular target could be useful tools for the imaging of overexpressed receptors in tumors. In this study, we aimed to develop radiobrominated PDGFRβ ligands and evaluate their effectiveness as PDGFRβ imaging probes. The radiolabeled ligands were designed by modification of 1-{2-[5-(2-methoxyethoxy)-1*H*- benzo[*d*]imidazol-1-yl]quinolin-8-yl}piperidin-4-amine (**1**), which shows selective inhibition profile toward PDGFRβ. The bromine atom was introduced directly into C-5 of the quinoline group of **1**, or indirectly by the conjugation of **1** with the 3-bromo benzoyl group. [^77^Br]1-{5-Bromo-2-[5-(2-methoxyethoxy)-1*H*-benzo[*d*]imidazol-1-yl]quinoline-8-yl}piperidin-4-amine ([^77^Br]**2**) and [^77^Br]-*N*-3-bromobenzoyl-1-{2-[5-(2-methoxyethoxy)-1*H*-benzo[*d*]imidazol-1-yl]quinolin-8-yl}-piperidin-4-amine ([^77^Br]**3**) were prepared using a bromodestannylation reaction. In a cellular uptake study, [^77^Br]**2** and [^77^Br]**3** more highly accumulatd in BxPC3-luc cells (PDGFRβ-positive) than in MCF7 cells (PDGFRβ-negative), and their accumulation was significantly reduced by pretreatment with inhibitors. In biodistribution experiments, [^77^Br]**2** accumulation was higher than [^77^Br]**3** accumulation at 1 h postinjection. These findings suggest that [^76^Br]**2** is more promising for positron emission tomography (PET) imaging of PDGFRβ than [^76^Br]**3**.

## Introduction

Receptor tyrosine kinases (RTKs) regulate cell differentiation, survival, migration, proliferation, metabolism, and angiogenesis, and their upregulation leads to uncontrollable cellular signaling in cancer^1–3^. Platelet-derived growth factor receptors (PDGFRs) are a family of RTKs, which when activated, trigger the phosphorylation of intracellular domain and activate the signaling pathway^4^. PDGFRα and PDGFRβ are two subtypes of PDGFRs^5^. Because PDGFRβ affects multiple tumors associated with various processes, including the autocrine growth stimulation of tumor cells and tumorigenesis, it has been targeted for the development of anticancer therapy^6^. Additionally, PDGFRβ expression can be a useful biomarker for the prediction of a cancer prognosis^7^, for example, a significant positive correlation between PDGFRβ expression and short overall survival (OS) in patients with angiosarcoma has been reported^8^. Therefore, the determination of PDGFRβ expression by noninvasive imaging is prominently meaningful in clinical oncology. For the development of PDGFRβ imaging probes, ligands with high affinity and good selectivity profile for PDGFRβ are desirable as carrier structures^9,10^.

Several approaches are available for the radiolabeling of bioactive molecules, and representative examples are as follows: (i) replacement of an element in the bioactive molecule with its radioactive isotope^11^; (ii) substitution of an element or a functional group in the bioactive molecule with another type of a radioactive element, such as a radiohalogen, or another functional group with chemical similarity containing a radionuclide^12^; and (iii) addition of a radioactive metal with a chelator^13,14^. Because most organic molecules contain C, N, and O elements, replacing one of these elements with ^11^C, ^13^N, or ^15^O can lead to the development of a radiotracer. This replacement normally does not change the biological activity of the original molecule, and allows for the study of the metabolism or pharmacokinetics of such molecules^12^. However, this type of tracer has limited use because of the short half-lives of ^11^C (t_1/2_ = 20 min), ^13^N (t_1/2_ = 10 min), and ^15^O (t_1/2_ = 2 min). Although the influence is generally smaller than radiometal incorporation, substitution with a radiohalogen could influence affinity for the molecular target, biodistribution, and metabolism of bioactive compounds^13^. Therefore, the choice of radionuclides and the introduced position should be optimized to develop radiotracers with a radiohalogen. ^18^F (t_1/2_ = 110 min) is frequently used as positron emission tomography (PET) imaging radionuclide. Labeling with fluorine normally requires a different method than labeling with other halogens, such as bromine and iodine because of the short half-life and distinct physical property of fluorine^12^. ^124^I has been an interesting radionuclide for clinical and experimental PET because of its relatively longer half-life (t_1/2_ = 4.2 day) and chemical properties, namely the same labeling methods for ^123^I and ^131^I, which have been frequently used in clinical nuclear medicine, can be available^12^. However, the decay properties of ^124^I are not ideal for PET because the positron abundance is only 23%. Meanwhile, radiobromine is not used as often as radiofluorine and radioiodine isotopes; however, in some cases, radiobromine shows characteristics superior to the other radiohalogens. ^76^Br is potentially useful for PET imaging because it decays with high positron abundance (55%)^15^. Although the high positron energy of ^76^Br (3.4 MeV) could be a disadvantage in terms of image resolution and absorbed radiation dose^16^, the relatively longer half-life (t_1/2_ = 16.1 h) of ^76^Br than those of ^11^C, ^13^N, ^15^O, and ^18^F could be advantageous. Values of physical properties of the brominated derivatives, such as stability, molecular size, lipophilicity, and solubility, range between those of fluorinated and iodinated derivatives^11^. Thus, the steric effect of radiobromine might be smaller than that of radioiodine^17^.

**Figure 1:**
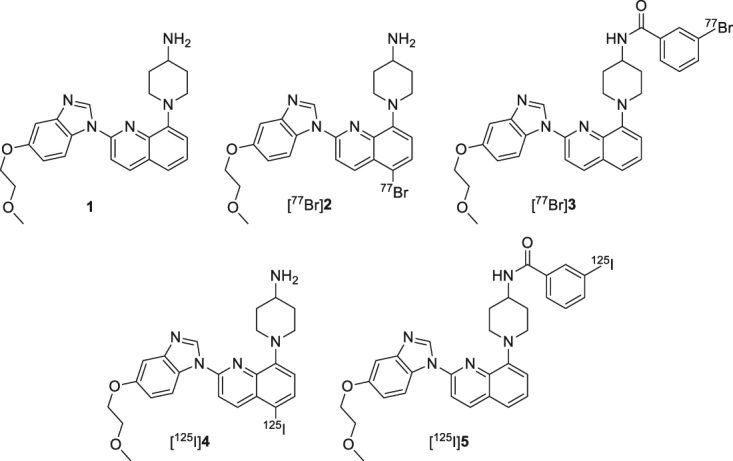
Chemical structures of **1**, [^77^Br]**2**, [^77^Br]**3**, [^125^I]**4**, [^125^I]**5**.

To date, several types of probes targeting PDGFRβ have been developed for cancer imaging in nuclear medicine. Askoxylakis *et al*. reported a ^125^I-labeled dodecapeptide targeting PDGFRβ with an inhibitory concentration 50 (IC_50_) value of 1.4 µM^9^. Tolmachev *et al*. reported PDGFRβ specific affibodies labeled with ^111^In or ^68^Ga, which exhibited a high tumor-to-blood ratio and good IC_50_. The labeled affibodies clearly visualized PDGFRβ-expressing U-87 MG xenografts in mice^18,19^. The peptide and affibodies bind to the extracellular part of PDGFRβ. Contrastingly, intracellular domains, especially adenosine triphosphate (ATP)-binding sites, could also be a promising target for PDGFRβ imaging. Radiolabeled tyrosine kinase inhibitors (TKIs), such as the ^11^C-labeled imatinib^20^, ^18^F-labeled dasatinib^21^, ^11^C-labeled sorafenib (tumor uptake in RXF393 xenograft mice about 2.52 ± 0.33%ID/g)^22^, and ^125^I-labeled sunitinib^23^, have been synthesized and evaluated in order to develop probes targeting the ATP-binding site in nuclear medicine. However, not only do they all bind PDGFRs, but also bind other RTKs, such as vascular endothelial growth factor receptors 2 (VEGFR2), BCR-ABL1, and c-KIT. Recently, we synthesized and evaluated radioiodinated **1** derivatives, [^125^I]1-{5-iodo-2-[5-(2-methoxyethoxy)-1*H*-benzo[*d*]imidazol-1-yl]quinolin-8-yl}piperidin-4-amine ([^125^I]**4**) and [^125^I]*N*-3-iodobenzoyl-1-{2-[5-(2-methoxyethoxy)-1*H*-benzo[*d*]imidazol-1-yl]quinolin-8-yl}-piperidin-4-amine ([^125^I]**5**) (Fig. 1), as PDGFRβ imaging probes^24^. In both *in vitro* and *in vivo* experiments, [^125^I]**4** showed a significantly higher tumor uptake with high PDGFRβ expression than [^125^I]**5**.

In this study, we describe the development of novel radiobrominated probes for PDGFRβ imaging. The strategy of these probes are similer to that of our previous developed radioiodine-labeled probes^12^, but differece between iodine and boromine should alter their characteristics and biodistribution of the probes^25–27^, and the difference of radionuclides may give much impact in clinical nuclear medicine. Two brominated **1** derivatives, 1-{5-bromo-2-[5-(2-methoxyethoxy)-1*H*-benzo[*d*]imidazol-1-yl]quinolin-8-yl}piperidin-4-amine (**2**) and *N*-3-bromobenzoyl-1-{2-[5-(2-methoxyethoxy)-1*H*-benzo[*d*]imidazol-1-yl]quinolin-8-yl}-piperidin-4-amine (**3**), were designed and synthesized. Bromine was incorporated into **1** instead of iodine in **4** and **5**, and their affinities for PDGFRβ were examined. Although we are interested in developing ^76^Br-labeled PDGFRβ PET imaging probes, ^77^Br was used in this initial study because it has a relatively longer half-life (t_1/2_ = 57 h) and using a different radioisotope of the same element in the chemical compound does not alter its overall biological profile. Radiobrominated compounds, [^77^Br]**2** and [^77^Br]**3** (Fig. 1), were synthesized and the *in vitro* and *in vivo* experiments were performed.

## Results

### Synthesis of the reference compounds and their precursors

**Figure 2:**
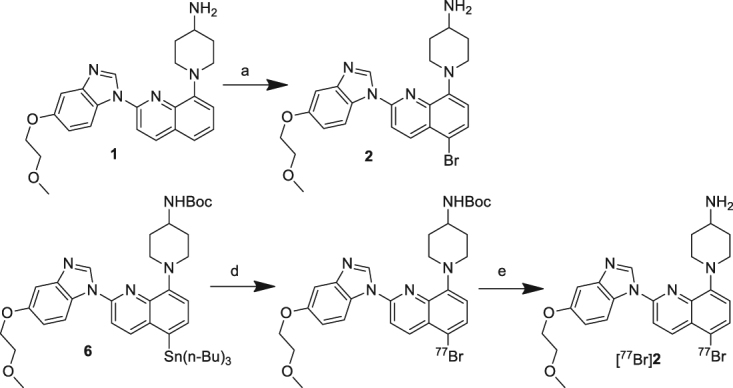
Synthesis of the reference and radiobrominated compounds of **2** (**a**) NBS, room temperature, overnight (**b**) [^77^Br]Br^−^, NCS, acetic acid, 60 °C, 30 min (**c**) TFA, room temperature, 30 min.

**Figure 3:**
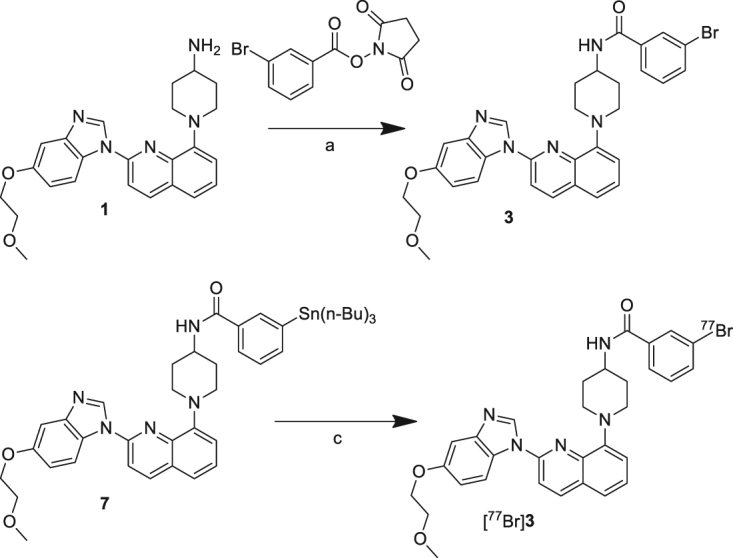
Synthesis of the reference and radioiodinated compounds of **3** (**a**) 50 °C, 2 h; (**b**) 50 °C, 3 h; (**c**) [^77^Br]Br^−^, NCS, acetic acid, room temperature, 15 min.

The nonradioactive brominated reference compounds, **2** and **3**, were synthesized as described in Figs 2 and 3. Compound **2** was synthesized by direct bromination at C-5 position of the quinoline group of **1** using *N*-bromosuccinimide (NBS) (Fig. 2). Compound **3** was obtained by acylation of the amino group of **1** using SBrB (Fig. 3). Tributyltin precursors (**6** and **7**) were synthesized as described previously^24^.

### Cell viability assays

**Figure 4:**
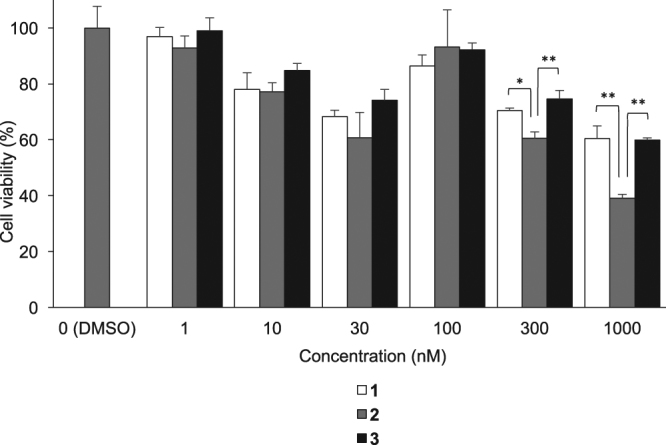
Cell viability after exposure **1**, **2**, and **3** by WST-8 assay. Data were presented as mean ± SD for three samples. Significance was determined using a one-way ANOVA followed by Tukey’s post hoc test (^*^p < 0.01, ^**^p < 0.001).

The binding affinity of **2** and **3** to the ATP-binding site in PDGFRβ was evaluated using PDGFRβ overexpressed TR-PCT1 cells. Cells were treated with 1–1000 nM of synthesized ligands, **1**, **2**, or **3**. As seen in Fig. 4, **3** showed the similar effects compared to **1**. **2** was more effective in decreasing the viability of TR-PCT1 cells than **1**.

### Radiolabeling

Radiobrominated compounds, [^77^Br]**2** and [^77^Br]**3**, were prepared in the condition without carrier addition using a bromodestannylation reaction of the corresponding tributyltin precursors (**6** and **7**) with high radiochemical yield (95 and 83%, respectively) (Figs 2 and 3). The effective molar activity of [^77^Br]**2** and [^77^Br]**3** was estimated to be approximately 2.0 × 10^12^ MBq/mol because the labeling used non-carrier-added condition, and the labeled compounds ([^77^Br]**2** and [^77^Br]**3**), and the precursors (**6** and **7**) were completely separated using RP-HPLC. *N*-chlorosuccinimide (NCS) was used as an oxidizing agent in these syntheses. The radiochemical purities of both radiotracers were over 99% after purification using RP-HPLC. The identity of [^77^Br]**2** and [^77^Br]**3** was verified by a retention time of the nonradioactive compounds **2** and **3** (Figs S1 and S2).

### Partition coefficient

The *n*-octanol/phosphate buffer partition coefficients of [^77^Br]**2** were 2.36 ± 0.06 which were lower than [^77^Br]**3** (3.02 ± 0.04). These values are lower than radioiodinated compounds, [^125^I]**4** and [^125^I]**5** at 2.71 ± 0.03 and 3.19 ± 0.17, respectively^24^.

### *In vitro* stability experiments

The stability of [^77^Br]**2** and [^77^Br]**3** in 0.1 M phosphate-buffered saline (PBS) (pH 7.4) was high (93.6 ± 0.9% and 93.0 ± 0.6%, respectively). There was no significant decomposition after 24 h incubation at 37 °C for either radiotracer.

### Cell uptake experiments

**Figure 5:**
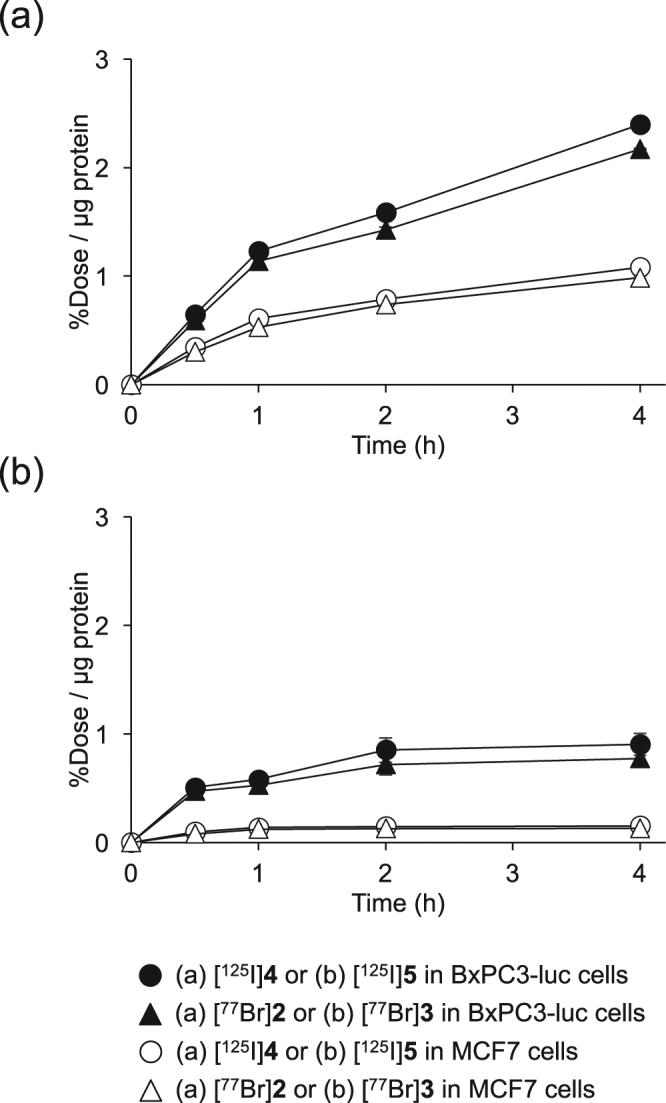
Cellular uptake study. Time-dependent accumulation of (**a**) [^77^Br]**2** and [^125^I]**4** (**b**) [^77^Br]**3** and [^125^I]**5** in BxPC3-luc and MCF7 cells. Data were presented as mean ± SD for three samples.

Figure 5 displays the cellular uptake of [^77^Br]**2** and [^77^Br]**3** into BxPC3-luc cells as PDGFRβ-positive and into MCF7 cells as PDGFRβ-negative^9,28,29^. The results demonstrated that the accumulation of coincubated [^77^Br]**2** and [^125^I]**4** in BxPC3-luc cells was higher than that in MCF7 cells BxPC3-luc cells showed a higher uptake of [^77^Br]**2** than that of [^77^Br]**3**. These results were consistent with the celluler uptake experiment of [^125^I]**4** and [^125^I]**5** using the same cell lines.

**Figure 6:**
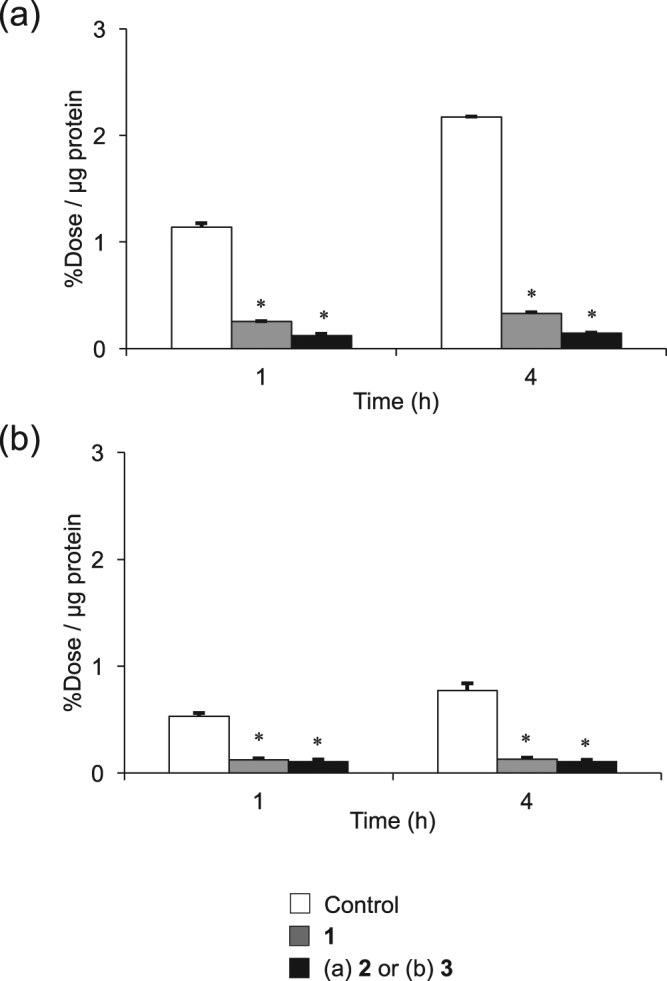
*In vitro* blocking studies of (**a**) [^77^Br]**2** and (**b**) [^77^Br]**3** in BxPC3-luc and MCF7 cells. Data were presented as mean ± SD for three samples. Significance was determined using a one-way ANOVA followed by Dunnett’s post hoc test (^*^p < 0.001, vs control [^77^Br]**2** or [^77^Br]**3**).

Blocking studies using **1**, **2**, and **3** were performed (Fig. 6). Pretreatment of an excess amount of **1** or **2** decreased uptakes of [^77^Br]**2** in BxPC3-luc cells. The uptake of [^77^Br]**3** was inhibited by a pretreatment using **1** or **3** in the same way.

### Competitive binding assay with BxPC3-luc cells

We investigated the competitive binding of [^125^I]**4** to BxPC3-luc cells with PDGFRβ ligand, **1**, **2**, and **3**. The IC_50_ values for **1**, **2**, and **3** were found to be 183 ± 8, 56 ± 13, and 1,283 ± 161 nM, respectively.

### Biodistribution experiments

In this study, to minimize both the number of mice consumed in the experiment and the potential for experimental errors, we co-injected radiobrominated and radioiodinated compounds into mice^30,31^. The biodistribution of [^77^Br]**2** and [^125^I]**4** in ddY mice were summarized in Table 1. Table 2 lists the biodistribution of [^77^Br]**3** and [^125^I]**5** in ddY mice. High radioactivity in the liver, small intestine, and large intestine was observed. At 24 h postinjection of the radiobrominated and radioiodinated compounds, radioactivity in feces was much higher than that in urine, suggesting hepatobiliary excretion as the main excretion pathway for both radiotracers.

**Table 1:** Biodistribution of radioactivity after administration co-injection of [^77^Br]**2** and [^125^I]**4** at 10 min, 1, 4, and 24 h intravenously in ddY mice.

Tissues	Time after injection			
	10 min	1 h	4 h	24 h
**[** ^**77**^ **Br]2**				
Blood	0.99 (0.03)	0.28 (0.06)	0.20 (0.03)	0.12 (0.02)
Liver	12.49 (0.64)	4.26 (1.37)	3.44 (0.98)	0.29 (0.08)
Kidney	15.61 (1.32)	10.12 (3.49)	4.33 (0.75)	0.21 (0.05)
Small intestine	8.58 (2.18)	29.51 (7.92)	4.76 (0.89)	0.07 (0.01)
Large intestine	0.99 (0.10)	2.69 (0.65)	53.70 (8.89)	0.18 (0.03)
Spleen	5.39 (0.40)	1.59 (0.28)	0.69 (0.24)	0.66 (0.77)
Pancreas	4.18 (0.38)	2.98 (0.75)	1.63 (0.19)	0.41 (0.34)
Lung	10.92 (0.55)	4.32 (0.84)	2.71 (1.06)	0.29 (0.05)
Heart	4.41 (0.27)	1.15 (0.17)	0.58 (0.13)	0.29 (0.03)
Stomach^†^	1.74 (0.15)	1.62 (0.55)	0.81 (0.21)	0.11 (0.01)
Bone	2.81 (1.06)	1.15 (0.15)	1.50 (1.27)	0.55 (0.10)
Muscle	2.24 (0.18)	0.92 (0.04)	0.44 (0.07)	0.22 (0.02)
Brain	0.12 (0.01)	0.10 (0.01)	0.09 (0.00)	0.07 (0.00)
Urine				2.82 (0.19)
Feces				68.62 (2.21)
**[** ^**125**^ **I]4**				
Blood	1.15 (0.08)^*^	0.32 (0.09)	0.19 (0.05)	0.03 (0.00)^**^
Liver	11.59 (0.69)	3.72 (1.18)	3.45 (1.02)	0.29 (0.08)
Kidney	14.44 (1.31)	8.18 (2.71)	4.28 (0.68)	0.13 (0.03)
Small intestine	9.32 (2.30)	25.44 (6.86)	4.98 (0.89)	0.04 (0.00)^**^
Large intestine	0.80 (0.07)^*^	2.16 (0.54)	51.43 (8.57)	0.14 (0.02)
Spleen	5.08 (0.56)	1.67 (0.41)	0.43 (0.11)	0.11 (0.09)^*^
Pancreas	3.52 (0.33)^*^	2.49 (0.63)	1.27 (0.11)^*^	0.07 (0.02)
Lung	9.64 (0.66)^*^	3.88 (0.72)	2.18 (0.55)	0.15 (0.08)^*^
Heart	4.27 (0.27)	1.23 (0.49)	0.31 (0.05)^*^	0.10 (0.09)^**^
Stomach^†^	1.66 (0.11)	1.76 (0.58)	0.85 (0.21)	0.05 (0.00)^**^
Bone	1.53 (0.14)	1.22 (0.29)	0.32 (0.08)	0.15 (0.05)^**^
Muscle	1.92 (0.18)	0.78 (0.11)	0.23 (0.06)^**^	0.04 (0.01)^**^
Brain	0.07 (0.00)^**^	0.15 (0.04)	0.03 (0.00)^**^	0.02 (0.00)^**^
Urine				2.51 (0.05)
Feces				66.62 (1.97)

Data were presented as %injected dose/gram tissue. Each value represent mean ± SD for four mice. Significance was determined using an unpaired Student’s *t*-test (^*^p < 0.05, ^**^p < 0.01 vs. [^125^I]**4**).

^†^presented as %injected dose /organ.

**Table 2:** Biodistribution of radioactivity after administration co-injection of [^77^Br]**3** and [^125^I]**5** at 10 min, 1, 4, and 24 h intravenously in ddY mice.

Tissues	Time after injection			
	10 min	1 h	4 h	24 h
**[** ^**77**^ **Br]3**				
Blood	1.68 (0.07)	0.65 (0.05)	0.25 (0.04)	0.39 (0.05)
Liver	25.80 (2.54)	7.59 (0.76)	2.19 (1.38)	0.18 (0.04)
Kidney	5.21 (0.53)	2.03 (0.33)	0.65 (0.15)	0.37 (0.05)
Small intestine	3.28 (0.32)	14.30 (0.23)	4.50 (2.02)	0.18 (0.05)
Large intestine	0.88 (0.08)	2.23 (0.75)	28.99 (2.21)	0.18 (0.00)
Spleen	3.05 (1.14)	1.47 (0.20)	0.88 (0.08)	0.72 (0.24)
Pancreas	2.61 (0.20)	1.24 (0.16)	0.68 (0.10)	0.68 (0.03)
Lung	3.08 (0.65)	1.18 (0.10)	0.45 (0.07)	0.56 (0.07)
Heart	2.70 (0.24)	1.14 (0.09)	0.65 (0.05)	0.72 (0.11)
Stomach^†^	1.31 (0.22)	1.10 (0.24)	0.59 (0.26)	0.35 (0.06)
Bone	1.88 (0.15)	1.60 (0.27)	1.32 (0.12)	1.32 (0.17)
Muscle	1.28 (0.19)	0.91 (0.45)	0.57 (0.24)	0.54 (0.04)
Brain	0.43 (0.02)	0.22 (0.05)	0.19 (0.01)	0.20 (0.02)
Urine				2.93 (0.25)
Feces				73.92 (1.35)
**[** ^**125**^ **I]5**				
Blood	0.94 (0.14)^**^	0.26 (0.05)^**^	0.08 (0.01)^**^	0.01 (0.00)^**^
Liver	16.37 (2.05)^**^	3.32 (0.49)^**^	1.21 (0.96)	0.06 (0.00)^**^
Kidney	5.23 (0.65)	1.57 (0.42)	0.36 (0.10)^*^	0.05 (0.00)^**^
Small intestine	9.59 (1.71)^**^	24.03 (1.45)^**^	1.32 (0.66)^*^	0.01 (0.00)^**^
Large intestine	0.75 (0.06)^*^	3.46 (2.18)	18.55 (1.85)^**^	0.02 (0.00)^**^
Spleen	1.72 (0.23)	0.70 (0.16)^**^	0.27 (0.09)^**^	0.02 (0.00)^**^
Pancreas	2.41 (0.20)	0.86 (0.07)^**^	0.27 (0.07)^**^	0.02 (0.00)^**^
Lung	1.80 (0.30)^*^	1.07 (0.08)	0.50 (0.19)	0.01 (0.00)^**^
Heart	1.66 (0.23)^**^	0.39 (0.15)^**^	0.20 (0.04)^**^	0.02 (0.01)^**^
Stomach^†^	2.09 (1.12)	0.78 (0.19)	0.27 (0.10)	0.01 (0.00)^**^
Bone	0.81 (0.11)^**^	0.64 (0.31)^***^	0.40 (0.09)^**^	0.06 (0.00)^**^
Muscle	0.97 (0.16)^*^	0.31 (0.13)^*^	0.16 (0.08)^*^	0.01 (0.00)^**^
Brain	0.19 (0.03)^**^	0.07 (0.03)^**^	0.01 (0.00)^**^	0.00 (0.00)^**^
Urine				12.36 (0.91)^**^
Feces				60.96 (5.80)^*^

Data were presented as %injected dose/gram tissue. Each value represent mean ± SD for four mice. Significance was determined using an unpaired Student’s *t*-test (^*^p < 0.05, ^**^p < 0.01 vs. [^125^I]**5**).

^†^presented as %injected dose /organ.

We investigated the biodistribution of the radiotracers in BxPC3-luc tumor-bearing mice by co-injecting [^77^Br]**2** with [^125^I]**4** (Table 3), and [^77^Br]**3** with [^125^I]**5** (Table 4). Accumulation of [^77^Br]**2** in the tumor at 1 h after administraion was 1.61% injected dose (ID)/g, which was significantly higher than that of [^77^Br]**3** (1.15%ID/gram).

**Table 3:** Biodistribution of radioactivity after administration co-injection of [^77^Br]**2** and [^125^I]**4** at 1 h intravenously in BxPC3-luc tumor-bearing mice.

Tissues	[^77^Br]**2**	[^125^I]**4**
Blood	0.58 (0.10)	0.63 (0.05)
Liver	11.07 (1.04)	11.02 (0.73)
Kidney	17.39 (4.03)	15.56 (3.61)
Small intestine	51.52 (9.93)	47.01 (5.56)
Large intestine	3.22 (0.40)	2.46 (0.35)
Spleen	2.94 (0.54)	2.76 (0.73)
Pancreas	6.61 (1.00)	5.48 (0.93)
Lung	10.31 (1.91)	9.14 (2.14)
Heart	1.88 (0.33)	1.51 (0.40)
Stomach^†^	0.96 (0.20)	0.97 (0.19)
Bone	1.15 (0.09)	0.83 (0.10)^*^
Muscle	0.94 (0.08)	0.79 (0.10)
Brain	0.14 (0.03)	0.07 (0.00)
BxPC3-luc tumor	1.61 (0.24)	1.19 (0.06)^*^

Data were presented as means ± SD of %injected dose/gram of tissue for three mice. Significance was determined using an unpaired Student’s *t*-test (^*^p < 0.05, ^**^p < 0.01 vs. [^125^I]**4**).

^†^presented as %injected dose /organ.

**Table 4:** Biodistribution of radioactivity after administration co-injection of [^77^Br]**3** and [^125^I]**5** at 1 h intravenously in BxPC3-luc tumor-bearing mice.

Tissues	[^77^Br]**3**	[^125^I]**5**
Blood	1.16 (0.02)	0.23 (0.02)^**^
Liver	19.82 (2.48)	4.83 (0.71)^**^
Kidney	5.95 (0.16)	1.13 (0.11)^**^
Small intestine	32.72 (3.10)	46.95 (2.88)^**^
Large intestine	5.53 (0.96)	7.98 (3.79)
Spleen	2.51 (0.17)	0.61 (0.06)^**^
Pancreas	2.15 (0.13)	1.25 (0.17)^**^
Lung	3.02 (0.51)	1.16 (0.23)^**^
Heart	1.83 (0.04)	0.42 (0.05)^**^
Stomach^†^	0.67 (0.14)	0.27 (0.06)^*^
Bone	1.32 (0.08)	0.31 (0.04)^**^
Muscle	1.05 (0.18)	0.38 (0.06)^**^
Brain	0.17 (0.02)	0.05 (0.00)^**^
BxPC3-luc tumor	1.15 (0.35)	0.55 (0.09)^*^

Data were presented as means ± SD of %injected dose/gram of tissue for three mice. Significance was determined using an unpaired Student’s *t*-test (^*^p < 0.05, ^**^p < 0.01 vs. [^125^I]**5**).

^†^presented as %injected dose /organ.

**Figure 7:**
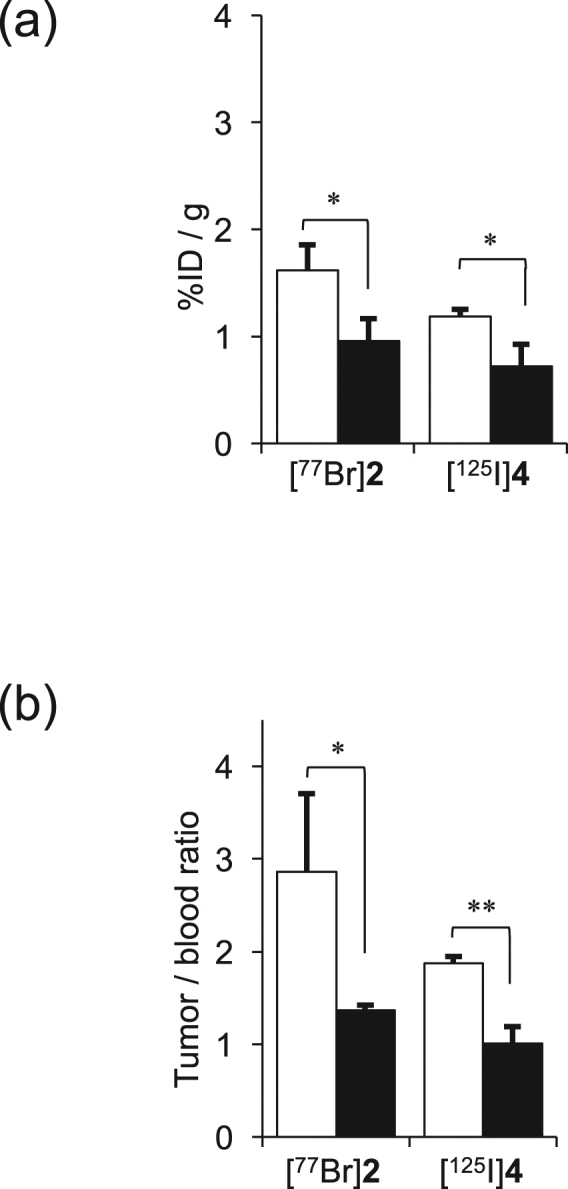
(**a**) Tumor uptake and (**b**) tumor/blood uptake ratios of [^77^Br]**2** and [^125^I]**4** at 1 h postinjection with **1** (40 mg/kg) (blocking group) or no treatment control group. Data were presented as means ± SD for three or four mice. Significance was determined using an unpaired Student’s *t*-test (^*^p < 0.05, ^**^p < 0.01).

Figure 7 displays the blocking effect of the pretreatment with **1** on the tumor accumulation of [^77^Br]**2** at 1 h postinjection. The tumor uptake of both the control and blocking groups was 1.61 ± 0.24 and 0.96 ± 0.20% ID/g, respectively. Thus, the figures are shown as not only % ID/g but also as tumor-to-blood ratio. The pretreatment with **1** significantly reduced the tumor uptake and the tumor-to-blood ratio of [^77^Br]**2** at 1 h postinjection.

## Discussion

In our study, we performed cell viability assays of **1**, **2**, and **3**, to evaluate the effect of the structural changes on the affinity between the ligand and the molecular target, PDGFRβ. Similar affinity was exhibited by **1** and **3**, whereas **2** displayed a higher affinity than **1** (Fig. 4). The larger size of the bromine compared with hydrogen may have contributed to this result. In accordance with **4**, incorporating bromine into **1** could increase the affinity of **2** for PDGFRβ. Although the results of the cell viability assays showed the *in vitro* affinity of the iodinated compound **4** was higher than that of the brominated compound **2**, the competitive binding assay using BxPC3-luc cells showed the affinity of **2** was comparable to that of **4**.

The comparison of chloramine-T, peracetic acid, and NCS in this study showed NCS was the best oxidizing agent for the bromination of **1** through an oxidative bromodestannylation reaction under non-carrier added condition (data not shown). When NCS was used, undesired radioactive peaks had almost disappeared. Previously, we reported the preparation of a ^77^Br-labeled sigma-1 receptor ligand, (+)-[^77^Br]*p*BrV, by using oxidative bromodestannylation with chloramine-T; its radiochemical yield was 53%^30^. Hanaoka *et al*. performed ^77^Br labeling of α-methyl-phenylalanine by bromodestannylation reaction with NCS, and its radiochemical yield was approximately 60%^32^. In these reports, using the same corresponding precursors, the radiochemical yields for radiobromine labeling were lower than those for radioiodine labeling. In this study, we obtained [^77^Br]**2** and [^77^Br]**3** with prominently high radiochemical yields (95% and 83%, respectively), and the radiochemical yields were comparable to those of the corresponding radioiodinated compounds at 95% for [^125^I]**4** and 85% for [^125^I]**5**^24^.

In cellular uptake experiments for [^77^Br]**2** and [^77^Br]**3**, both radiolabeled compounds more highly accumulated in BxPC3-luc cells than in MCF7 cells, and [^77^Br]**2** showed higher accumulation in BxPC3-luc cells (PDGFRβ-positive) than [^77^Br]**3** (Fig. 5). This result was consistent with the cell viability assay, in which **2** showed a higher affinity for PDGFRβ than **1** and **3**. This result also agreed with the *in vivo* experiment in which [^77^Br]**2** showed higher accumulation in the BxPC3-luc tumor than [^77^Br]**3** (Table 2). The difference in the lipophilicity may be an important factor that contributed to this result. Moreover, the excess amount of PDGFRβ ligand can reduce [^77^Br]**2** uptake in PDGFRβ-positive tumor cells (Fig. 6) and in the *in vivo* blocking experiments using tumor-bearing mice (Fig. 7). [^77^Br]**2** uptake in tumors should be PDGFRβ specific, and [^77^Br]**2** should bind to the ATP-binding site of PDGFRβ in the tumor cell.

Although free iodide ions generated by the deiodination of the radioiodine-labeled compounds highly accumulate in the stomach and thyroid. However, the biodistribution of free bromide ions is much different. Because the free bromide ions accumulate in blood and are retained for a long time^32,33^, the radioactivity in the blood can be used as an *in vivo* stability index for radiobromine-labeled compounds. As summarized in Tables 1 and 2, [^77^Br]**2** and [^77^Br]**3** were stable *in vivo* because the blood clearances of both radiotracers were rapid. However, compared with [^77^Br]**2**, [^77^Br]**3** may have a slightly lower *in vivo* stability because the radioactivity in blood after the [^77^Br]**3** injection was slightly higher than that after the [^125^I]**5** injection in double-tracers biodistribution experiments. In contrast, radioactivity in the blood after the [^77^Br]**2** injection was almost the same as that after the [^125^I]**4** injection.

The present data, obtained from tumor bearing mice (Tables 3 and 4), showed that the tumor accumulations of both radiobrominated compounds, [^77^Br]**2** and [^77^Br]**3**, are greater than those of corresponding radioiodinated compounds, [^125^I]**4** and [^125^I]**5**. Lower lipophilicity and/or smaller molecule size of the radiobrominated compounds compared with the corresponding radioiodinated compounds might contribute to these results. The tumor-to-blood ratio of radioactivity at 1 h postinjection was 2.8 for [^77^Br]**2** and 1.9 for [^125^I]**4**, indicating that the radiobrominated **1** derivatives are more promising than radioiodinated **1** derivatives. However, the tumor uptake of a radiobrominated compound was not high enough as an appropriate probe for PDGFRβ imaging, and further modification is still needed.

In conclusion, [^77^Br]**2** and [^77^Br]**3** were easily prepared using a bromodestannylation reaction without carrier addition in excellent radiochemical yields and high radiochemical purities. Furthermore, ^76^Br could be incorporated into **1** instead of ^77^Br. Although this study suggests that radiobrominated **2** has more promising property for PET imaging of PDGFRβ than radioiodinated **4**, in clinical application of the radiobrominated compound as a PDGFRβ-targeted PET imaging agent, structural modification would be required to improve tumor uptake and tumor-to-background ratios.

## Methods

### Materials

Commercialy available reagents and solvents were purchased from Nacalai Tesque, Inc., (Kyoto, Japan), Wako Pure Chemical Industries (Osaka, Japan), Tokyo Chemical Industry Co., Ltd., (Tokyo, Japan), Sigma-Aldrich (St. Louis, MO, USA), and Kanto Chemical, Co., Inc. (Tokyo, Japan) and used without further purification.

The radioactivity was determined by a gamma counter (AccuFLEX γ ARC-8001 Hitachi, Ltd., Tokyo, Japan).

Bicinchoninic Acid (BCA) Protein Assay Kit was purchased from Nacalai Tesque. Cell Counting Kit-8 (CCK-8) was purchased from Dojindo (Kumamoto, Japan). Recombinant murine platelet-derived growth factor-BB (PDGF-BB) was purchased from PeproTech (Rocky Hill, NJ, USA). TR-PCT1 rodent brain pericyte cell line was generously obtained from Dr. Emi Nakashima (Keio University, Tokyo, Japan)^34^. BxPC3-luc cell line was obtained from JCRB Cell Bank (Ibaraki, Japan). MCF7 cell line was purchased from DS Pharma Biomedical (Osaka, Japan).

Proton and carbon nuclear magnetic resonance (^1^H-NMR and ^13^C-NMR) spectra were recorded on JEOL JNM-ECS400 (JEOL Ltd, Tokyo, Japan). Pictures of the NMR spectra are available in the Supplementary Material file. Direct analysis in real time mass spectra (DART-MS) and Electrospray ionization mass spectra (ESI-MS) were obtained with JEOL JMS-T100TD (JEOL Ltd). Purification was performed using HPLC system (LC-20AD pump, SPD-20A UV detector, and CTO-20A column oven, and DGU-20A_5R_ degasser, SHIMADZU, Kyoto, Japan). TLC analyses were performed with silica plates (Art 5553, Merck, Darmstadt, Germany). Optical density in WST-8 assay was determined using Infinite^®^ F200 Pro microplate reader (TECAN, Männedorf, Switzerland).

### Synthesis of reference compounds and precursors

Intermediate, reference compounds, **1**, and corresponding tin precursors were synthesized according to the reported studies, with a slight modification^24^.

### 1-{5-Bromo-2-[5-(2-methoxyethoxy)-1*H*-benzo[*d*]imidazol-1-yl]quinolin-8-yl}piperidin-4-amine (2)

**1** (144 mg, 0.4 mmol, 1.0 eq.) was dissolved in acetic acid (5 mL). To the solution, NBS (72 mg, 0.4 mmol, 1.0 eq.) was added in one portion and the mixture was stirred overnight at room temperature. pH was adjusted to 9.0 with saturated aqueous NaHCO_3_ and the mixture was extracted using dichloromethane (DCM) (25 × 3 mL). Organic phases were combined, dried over Na_2_SO_4_, filtered, and concentrated in vacuo. The residue was purified with SiO_2_ column chromatography (eluent: chloroform/methanol = 50/1) to afford **2** (169 mg, 85%) as a pale yellow solid. The brominated position of **2** was identified by NMR (^1^H, ^13^C, ^1^H-^1^H COSY, ^1^H-^13^C HMBC, and ^1^H-^13^C HMQC). ^1^H NMR (400 MHz, CDCl_3_): δ 1.81–1.83 (2H, m), 2.06–2.09 (2H, m), 2.87–2.93 (3H, m), 3.50 (3H, s), 3.83–3.84 (2H, m), 3.88 (2H, br d), 4.23–4.25 (2H, m), 7.12 (1H, d, J = 8.4 Hz), 7.17 (1H, dd, J = 8.8, 2.0 Hz), 7.37 (1H, d, J = 2.4 Hz), 7.71 (1H, dd, J = 8.0, 2.8 Hz), 7.78 (1H, d, J = 8.8 Hz), 8.41 (1H, d, J = 8.8 Hz), 8.68 (1H, d, J = 2.0 Hz), 8.71 (1H, dd, J = 8.8, 2.0 Hz). ^13^C NMR (100 MHz, CDCl_3_): δ 156.08, 149.44, 146.99, 145.51, 142.33, 141.36, 139.96, 130.16, 126.75, 126.69, 118.69, 114.96, 114.42, 113.99, 113.21, 103.53, 71.10, 67.78, 59.27, 51.46 (2C), 48.78, 35.96 (2C). LRMS (DART+): m/z (rel. intensity) = 496.1 (100) [M(^79^Br) + H^+^], 498.1 (98) [M(^81^Br) + H^+^].

### N-3-bromobenzoyl-1-{2-[5-(2-methoxyethoxy)-1*H*-benzo[*d*]imidazol-1-yl]-quinolin-8-yl}-piperidin-4-amine (3)

A mixture of **1** (15 mg, 37.5 µmol, 1.0 eq.), N,N-diisopropylethylamine (DIPEA) (10 µL, 56.2 µmol, 1.5 eq.), and N-succinimidyl-3-bromobenzoate (SBrB) (12 mg, 41.2 µmol, 1.1 eq.) in anhydrous DCM (1 mL) was stirred at 50 °C for 2 h under N_2_ atmosphere. Then the mixture was diluted with DCM (15 mL), washed with water (3 × 15 mL), dried over Na_2_SO_4_, filtered, and concentrated in vacuo. The residue was purified by SiO_2_ column chromatography (eluent: chloroform/methanol = 100/1) to yield **3** (18 mg, 80%) as a colorless solid. ^1^H-NMR (400 MHz, DMSO-d_6_): δ 2.01–2.08 (4H, m), 2.84–2.89 (2H, m), 3.31 (3H, s), 3.68–3.71 (2H, m), 3.87 (2H, br d), 4.02 (1H, br s), 4.19–4.21 (2H, m), 7.31–7.38 (4H, m), 7.52 (1H, t, J = 8.0 Hz), 7.65 (1H, d, J = 8.4 Hz), 7.92 (2H, t, J = 8.0 Hz), 8.19 (1H, d, J = 8.8 Hz), 8.24 (1H, s), 8.56 (1H, d, J = 8.4 Hz), 8.64 (1H, d, J = 8.0 Hz), 8.95 (1H, d, J = 8.8 Hz), 9.19 (1H, s). ^13^C NMR (100 MHz, CDCl_3_): δ 164.37, 155.54, 149.05, 146.96, 145.11, 142.41, 140.49, 140.32, 137.10, 133.84, 130.55, 129.94, 127.33, 126.62, 126.38, 126.30, 121.60 (2C), 118.33, 116.34, 114.19, 112.44, 102.77, 70.49, 67.25, 58.17, 51.35 (2C), 47.19, 32.09 (2C). LRMS (DART + ): m/z (rel. intensity) = 600.2 (100) [M(^79^Br) + H^+^], 602.2 (94) [M(^81^Br) + H^+^].

### Cell viability assays

The cell viability assay of brominated compounds, **2** and **3**, was evaluated as described previously^24,27^. Namely, TR-PCT1 cells were seeded on 96-well plates (5 × 10^3^ cells/well) and cultured at 33 °C in DMEM medium with 20 ng/mL PDGF-BB and 2% FBS in a 5% CO_2_ incubator. Cells were treated with each compound for 72 h and cell viability was determined by the Cell Counting Kit-8.

### Production of bromine-77

^77^Br was produced at University of Fukui. Radiosynthesis isolation and purification of ^77^Br were performed according to a previously reported method from a ^77^Se(p,n)^77^Br reaction on an isotopically enriched Cu_2_^77^Se coated tungsten target with 8 µA/11 MeV proton beam on a RDS Eclipse HP/RD cyclotron (Siemens, Knoxville, TN, USA)^30^.

### Radiolabeling

Radiotracers, [^77^Br]**2** and [^77^Br]**3**, were prepared by a bromodestannylation reaction using the corresponding tin precursors (**6** or **7**) and NCS as an oxidizing agent. The radiolabeled compounds were purified by reversed phase (RP)-HPLC performed with a Cosmosil 5C_18_-MS-II column (4.6 × 150 mm; Nacalai Tesque) at the flow rate of 1 mL/min with a gradient mobile phase of 70% methanol in water with 0.05% TEA to 90% methanol in water with 0.05% TEA for 20 min. The column temperature was 40 °C. Radiochemical yield and radiochemical purity were calculated by counting radioactivity using an auto well gamma counter.

### Synthesis of [^77^Br]2

A mixture of **6** (1 mg/mL, 5 µL), acetic acid (5%, 30 µL), acetonitrile (55 µL), and NCS (5 mg/mL, 10 µL) was charged into a sealed vial containing [^77^Br]Br^−^ (non-carrier added, 370 kBq). The mixture was heated to 60 °C for 30 min and shaken every 10 min during heating, then quenched by addition of sodium hydrogensulfite (5 mg/mL, 10 µL), and the solvent was removed by N_2_ gassing. Trifluoroacetic acid (TFA) was added to the residue and the shaking was allowed to continue for 30 min. After removing TFA by N_2_ gassing, the residue was mixed with the initial mobile phase of HPLC. The reaction mixture was shaken for some minutes, filtered, and purified by HPLC.

### Synthesis of [^77^Br]3

A mixture of **7** (1 mg/mL, 5 µL), acetic acid (5%, 15 µL), and NCS (5 mg/mL, 10 µL) was charged into a sealed vial containing [^77^Br]Br^−^ (non-carrier added, 370 kBq). The mixture was heated to 60 °C for 25 min and shaken every 10 min during heating and purified by HPLC.

### Determination of partition coefficients

Partition coefficients of [^77^Br]**2** and [^77^Br]**3** into n-octanol and 0.1 M phosphate buffer (PB) pH 7.4 were determined using the method described previously^24^. The measurement was performed in quadruplicate. The partition coefficient was determined by calculating the ratio of cpm/mL of n-octanol to that of buffer and expressed as log P. Radioactivity of each layer were counted by a gamma counter.

### *In vitro* stability experiments

The stability of radiolabeled compounds, [^77^Br]**2** and [^77^Br]**3**, were analyzed as described previously^24^. The purities radiolabeled compounds were determined by TLC using chloroform/methanol = 5/1 and 20/1 as a developing solvent for [^77^Br]**2** and [^77^Br]**3**, respectively. The results were confirmed by RP-HPLC.

### Cellular uptake experiments

Radiotracer uptake experiments in tumor cells were performed using BxPC3-luc and MCF7 cell lines. To compare the uptake between radiobrominated compounds, [^77^Br]**2** and [^77^Br]**3**, and the corresponding radioiodinated compounds, [^125^I]**4** and [^125^I]**5**, the cellular uptake experiments were performed by a double tracer method as described previously with a slight modification^30,31,35^. Briefly, the cells were seeded in RPMI 1640 medium containing 10% FBS and antibiotics on six-well plates (2 × 10^5^ cells/well) for 24 h in a 5% CO_2_ incubator at 37 °C. After removal of medium, cells were incubated in medium without FBS containing [^77^Br]**2** (3.7 kBq/well) and [^125^I]**4** (3.7 kBq/well) or [^77^Br]**3** (3.7 kBq/well) and [^125^I]**5** (3.7 kBq/well) and tween-80 (0.1%) for 0.5, 1, 2 and 4 h. Radioiodinated compounds were prepared as described previously]^24^. Cells were washed with ice-cold 1 mL of PBS and dissolved using 0.5 mL of 1 M NaOH and wells were washed with 1 M NaOH aqueous solution (0.5 mL). The radioactivity of pooled basic fractions was counted by a gamma counter. A range between 16 and 71 keV was used for measuring ^125^I and between 95 and 700 for ^77^Br. When radioactivity of ^77^Br was counted, the crossover of ^125^I activity into the ^77^Br channel was negligible. More than one month after the experiment, the radioactivity of ^125^I was determined because at that time ^77^Br has been decayed and its radioactivity was negligible. The protein in the cell was quantified by a BCA Protein Assay Kit. All data were expressed as %dose/µg protein.

For *in vitro* blocking experiment, inhibitor (**1**, **2**, or **3** with final concentration 10 µM) in 1 mL of medium without FBS was added to wells containing 2 × 10^5^ cells/well. After 10 min incubation, [^77^Br]**2** or [^77^Br]**3** (3.7 kBq/well) in 1 mL of medium without FBS was added to each well. Radioactivity and protein concentration in the cells were determined by the same method above-mentioned.

### Competitive binding assay using BxPC3-luc cells

BxPC3-luc cells in medium containing 10% FBS and antibiotics were seeded on 24-well plates (50,000 cells/wells) and incubated for 24 h in a 5% CO_2_ incubator at 37 °C. Nine concentrations of displacing nonradiolabeled ligands (**1**, **2** and **3**) (ranging from 1 pM to 1 mM) and [^125^I]**4** in medium without FBS were incubated at 37 °C for 4 h. After washing the cells twice using 250 µL of ice-cold PBS, the unbound radioligand was removed. The cells were dissolved using 250 µL of 1 M NaOH and wells were washed with 250 µL of 1 M NaOH. The bound radioactivity was determined using a gamma counter.

### Animals

Animal experiments were conducted in strict accordance with the Guidelines for the Care and Use of Laboratory Animals of Kanazawa University. The animal experimental protocols used were approved by the Committee on Animal Experimentation of Kanazawa University (Permit Number: AP-163766). The animals were housed with free access to food and water at approximately 23 °C with a 12 h light/dark schedule. ddY mice (six-week-old, male, 27–30 g) and BALB/c nu/nu mice (four-week-old, female, 12–17 g) were purchased from Japan SLC Inc. (Hamamatsu, Japan). For preparing the tumor-bearing mice, BxPC3-luc cells (5 × 10^6^ cells) were subcutaneously injected into the left shoulder of BALB/c nu/nu mice. The tumor reached palpable size after two weeks of the inoculation.

### Biodistribution experiments

Mice were intravenously injected via the tail with 100 µL of saline solution of [^77^Br]**2** and [^125^I]**4** or [^77^Br]**3** and [^125^I]**5** (74 kBq, respectively), containing 10% ethanol and 1% tween-80. The ddY mice were sacrificed at 10 min, 1, 4, and 24 h postinjection. Meanwhile, tumor-bearing mice were sacrificed at 1 h postinjection.

For *in vivo* blocking studies, the tumor-bearing mice were intraperitoneally injected with 200 µL of **1** (40 mg/kg in 15% ethanol and 85% water) 1 h before intravenous injection of 100 µL of saline solution containing [^77^Br]**2** and [^125^I]**4** or [^77^Br]**3** and [^125^I]**5** (74 kBq, respectively), 1% tween-80 and 10% ethanol. At 1 h postinjection of radiotracers, the mice were sacrificed.

Tissues in mice were resected and weighed. The radioactivity of the tissues was counted by a gamma counter and counts were corrected for background radiation. The data were expressed as percent injection dose per gram tissue (%ID/g).

### Statistical analysis

All data were statisticaly analyzed using GraphPad 5.0 software (La Jolla, CA, USA) and expressed as mean ± standard deviation (SD). Significance for *in vitro* blocking experiments was determined by a one-way analysis of variance (ANOVA) followed by Dunnett’s post hoc test compared to the control group. Significance in cell viability assays was determined by ANOVA followed by Tukey’s post hoc test. IC_50_ values for the binding assay were calculated by nonlinear regression. Significant differences in biodistribution experiments between [^77^Br]**2** and [^77^Br]**3** groups were determined using unpaired Student’s *t*-test. Significance for *in vivo* blocking studies between control and blocking groups were determined by unpaired Student’s *t*-test. Results were considered statistically significant at p < 0.05.

## Electronic supplementary material

Supplementary Information
