# Supplementary material for: Radiobrominated benzimidazole-quinoline derivatives as Platelet-derived growth factor receptor beta (PDGFRβ) imaging probes
Source: Sci Rep. 2018 Jul 10;8:10369. doi: 10.1038/s41598-018-28529-0 (PMC6039436; doi:10.1038/s41598-018-28529-0)

Supplementary Information

Radiobrominated benzimidazole-quinoline derivatives as Platelet-derived growth factor receptor beta (PDGFRβ) imaging probes

Nurmaya Effendi[a,b#], Kenji Mishiro[c#], Takeshi Takarada[d], Akira Makino[e], Daisuke Yamada[d], Yoji Kitamura[f], Kazuhiro Shiba[f], Yasushi Kiyono[e], Akira Odani[a], Kazuma Ogawa[a,c*]

[#]These authors contributed equally to this work

*Corresponding Author

Phone: 81-76-234-4460. Fax: 81-76-234-4459. E-mail: kogawa@p.kanazawa-u.ac.jp

Contents:

1. RP-HPLC chromatograms of **2** and [^{77}Br]**2** (**Figure S1**)
2. RP-HPLC chromatograms of **3** and [^{77}Br]**3** (**Figure S2**)
3. ^{1}H-NMR spectra of 1-{5-bromo-2-[5-(2-methoxyethoxy)-1*H*-benzo[*d*]imidazol-1-yl]quinolin-8-yl}piperidin-4-amine (**2**)
4. ^{13}C-NMR 1-{5-bromo-2-[5-(2-methoxyethoxy)-1*H*-benzo[*d*]imidazol-1-yl]quinolin-8-yl}piperidin-4-amine (**2**)
5. ^{1}H-^{1}H COSY 2D NMR 1-{5-bromo-2-[5-(2-methoxyethoxy)-1*H*-benzo[*d*]imidazol-1-yl]quinolin-8-yl}piperidin-4-amine (**2**)
6. ^{1}H-^{13}C HMBC 2D NMR 1-{5-bromo-2-[5-(2-methoxyethoxy)-1*H*-benzo[*d*]imidazol-1-yl]quinolin-8-yl}piperidin-4-amine (**2**)

7. ^{1}H-^{13}C HMQC 2D NMR 1-{5-bromo-2-[5-(2-methoxyethoxy)-1*H*-benzo[*d*]imidazol-1-yl]quinolin-8-yl}piperidin-4-amine (**2**)
8. ^{1}H-NMR *N*-3-bromobenzoyl-1-{2-[5-(2-methoxyethoxy)-1*H*-benzo[*d*]imidazol-1-yl]-quinolin-8-yl}-piperidin-4-amine (**3**)
9. ^{13}C-NMR *N*-3-bromobenzoyl-1-{2-[5-(2-methoxyethoxy)-1*H*-benzo[*d*]imidazol-1-yl]-quinolin-8-yl}-piperidin-4-amine (**3**)

1. HPLC chromatograms of **2** and [^{77}Br]**2**.

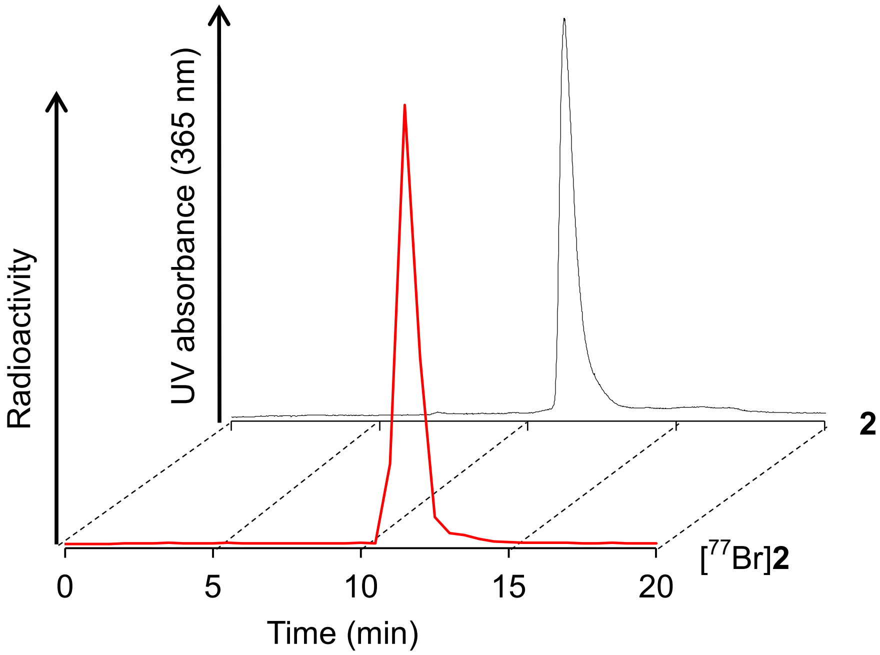

Figure S1. RP-HPLC chromatograms of **2** and [^{77}Br]**2**. Conditions: a Cosmosil 5C_{18}-MS-II column (4.6 ID × 150 mm) at the flow rate of 1 mL/min with a gradient mobile phase of 70 – 90% methanol in water with 0.05% TEA for 20 min. The column temperature was 40 °C.

2. HPLC chromatograms of [^{77}Br]**3** and **3**

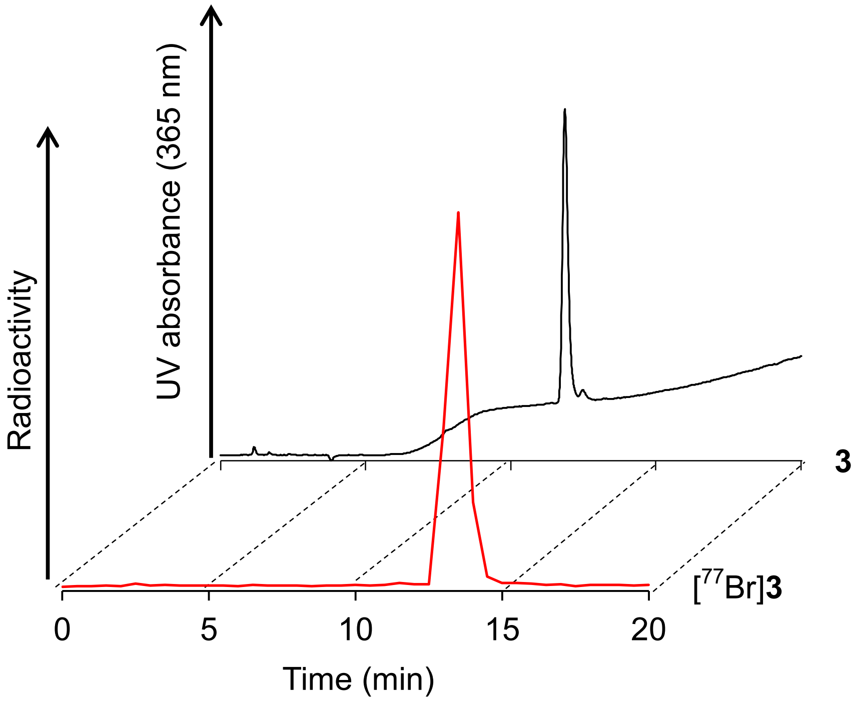

Figure S2. RP-HPLC chromatograms of **3** and [^{77}Br]**3**. HPLC conditions: a Cosmosil 5C$_{18}$-MS-II column (4.6 ID × 150 mm) at the flow rate of 1 mL/min with a gradient mobile phase of 70 – 90% methanol in water with 0.05% TEA for 20 min. The column temperature was 40 °C.

3. *^{1}H-NMR:* ***2*** *(CDCl$_3$)*

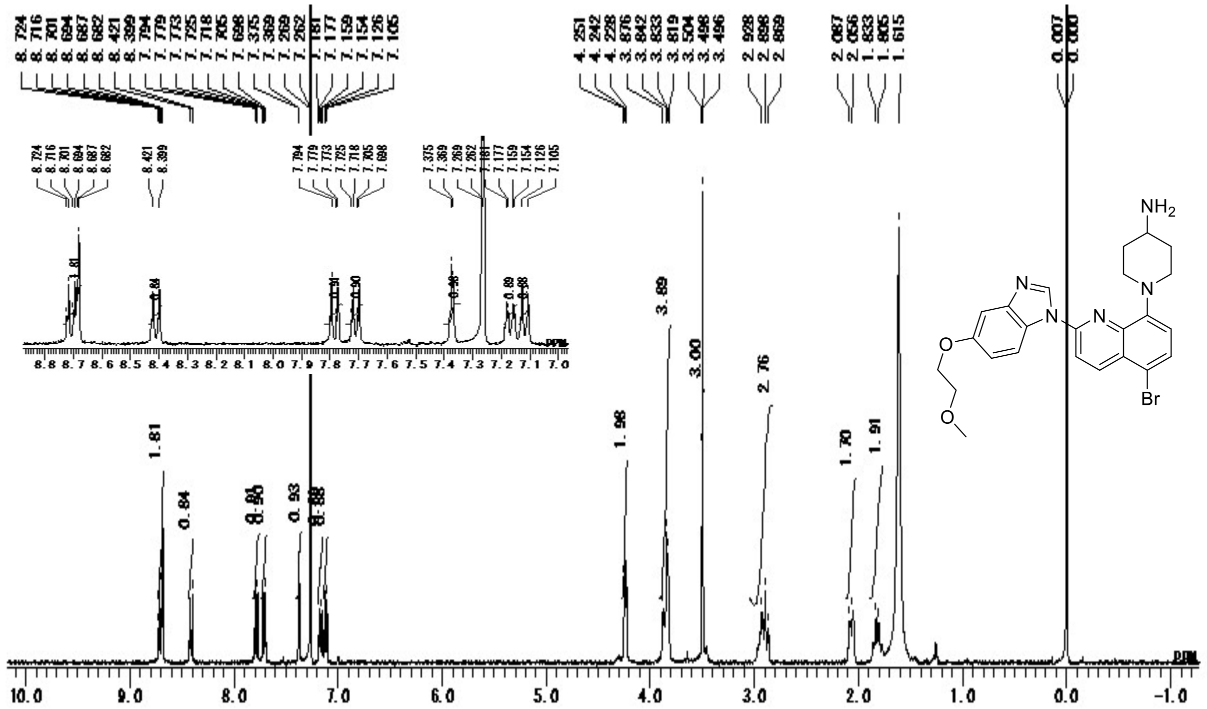

4. ^{13}C-NMR: **2** (CDCl$_3$)

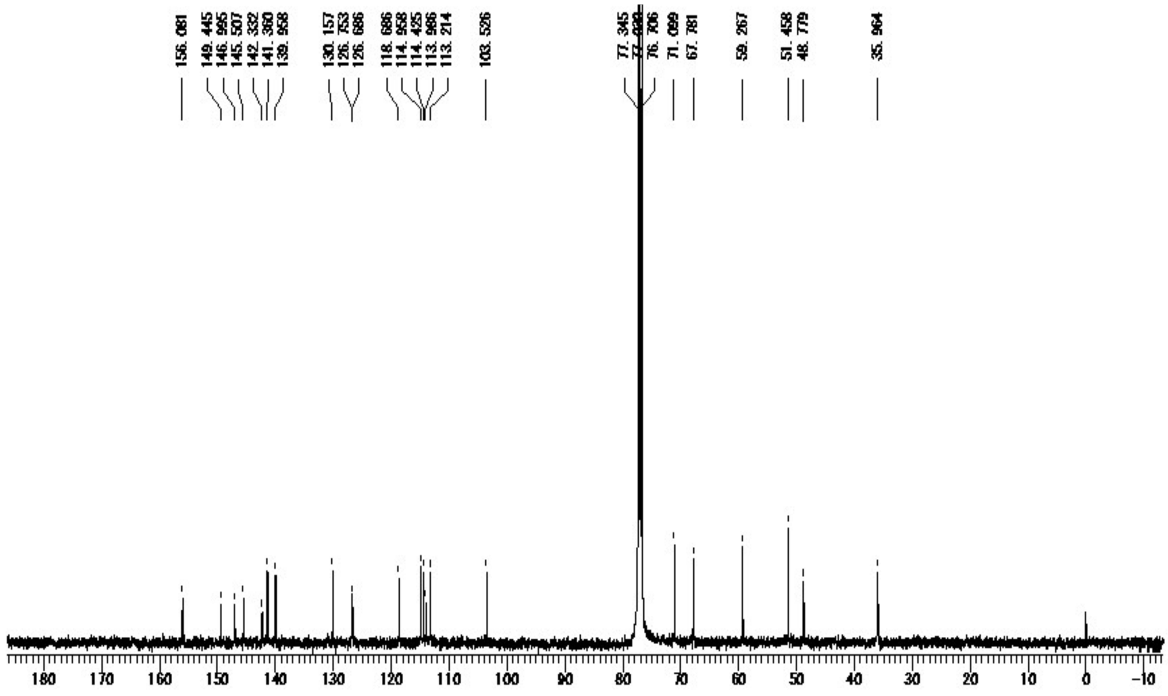

5. ^{1}H-^{1}H COSY 2D NMR: **2** ($CDCl_3$)

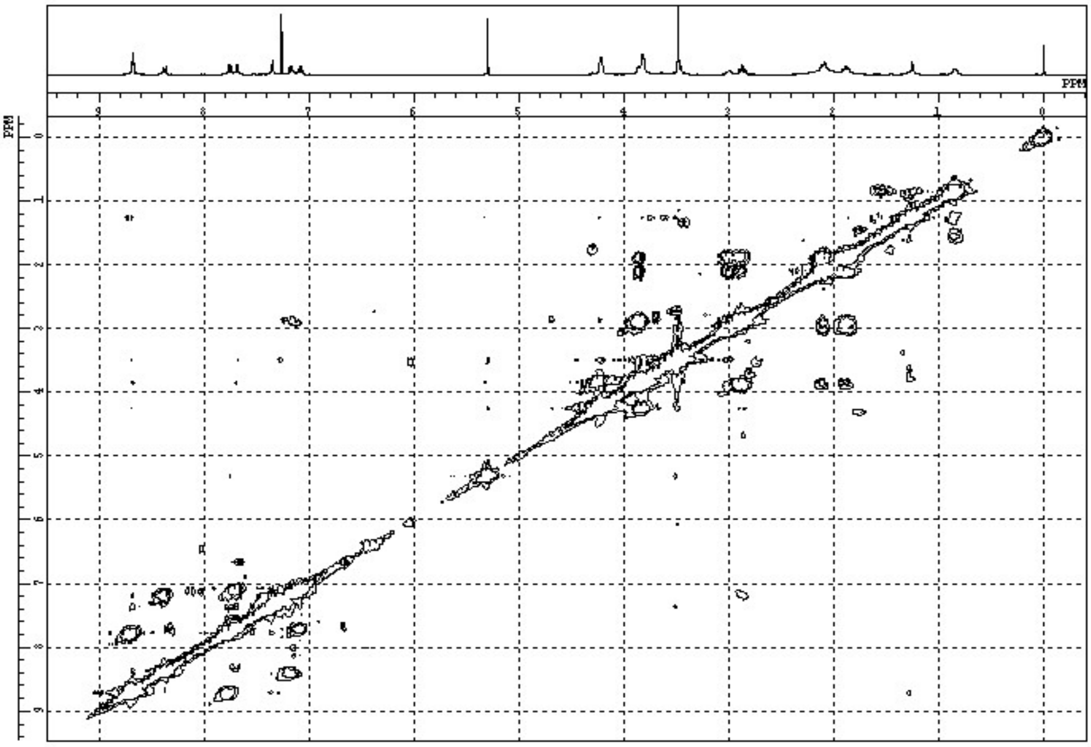

6. ^{1}H-^{13}C HMBC 2D NMR: **2** ($CDCl_3$)

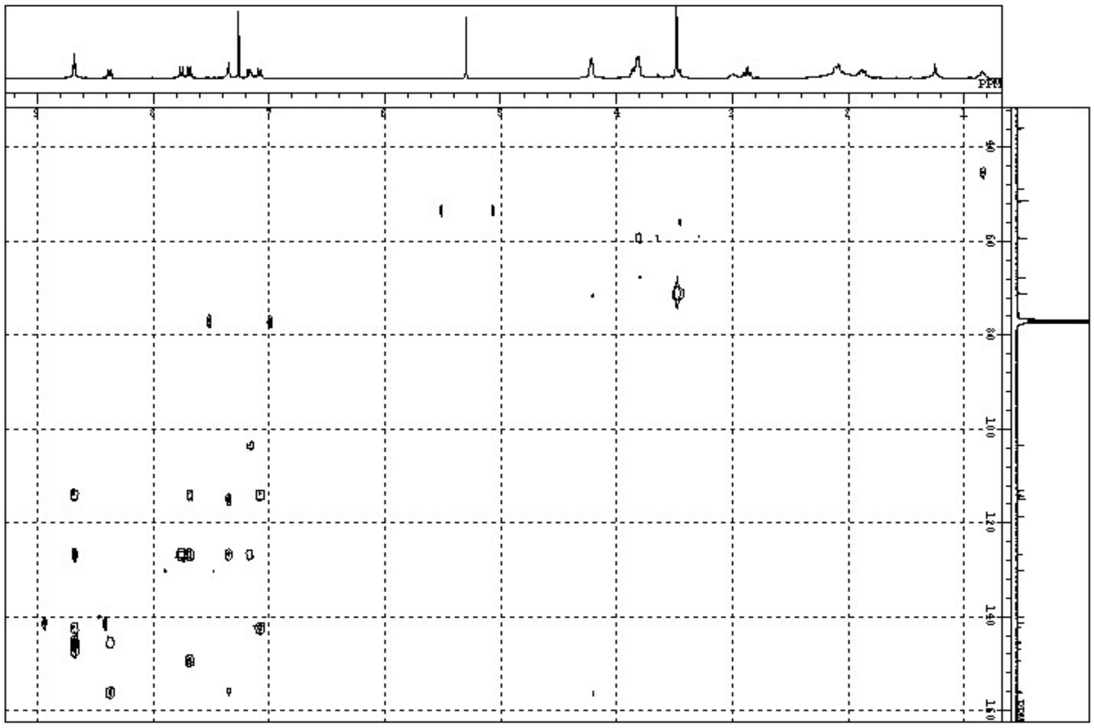

7. ^{1}H-^{13}C HMQC 2D NMR: **2** ($CDCl_3$)

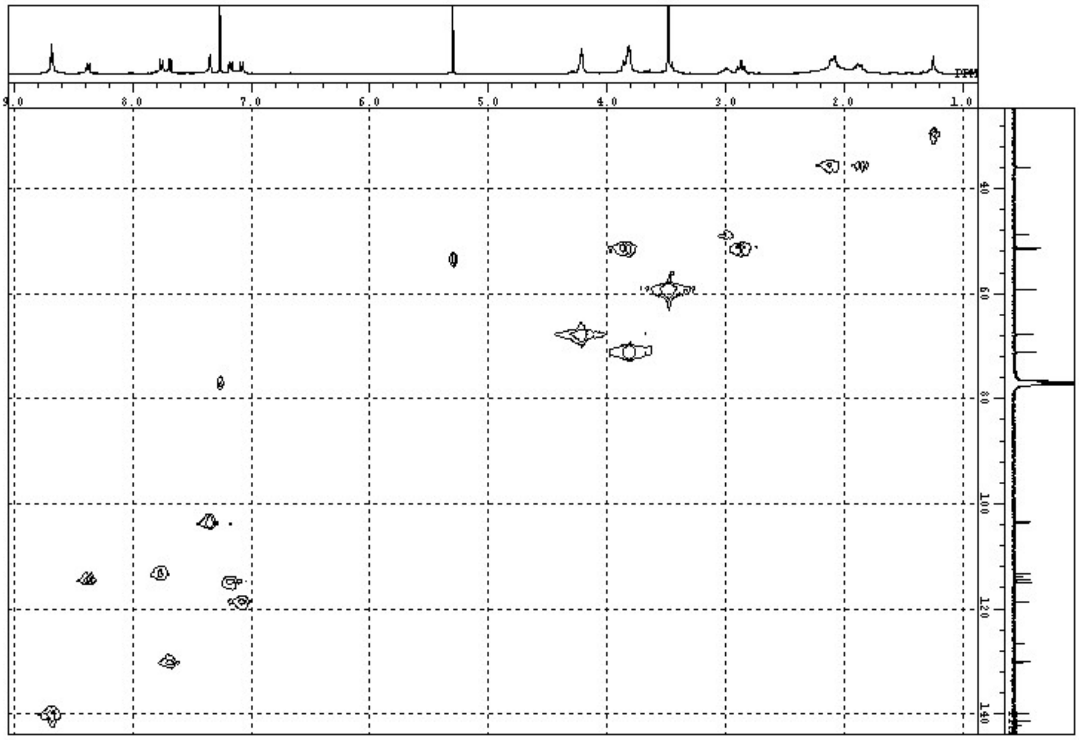

8. ^{1}H-NMR: **5** (DMSO-d_6)

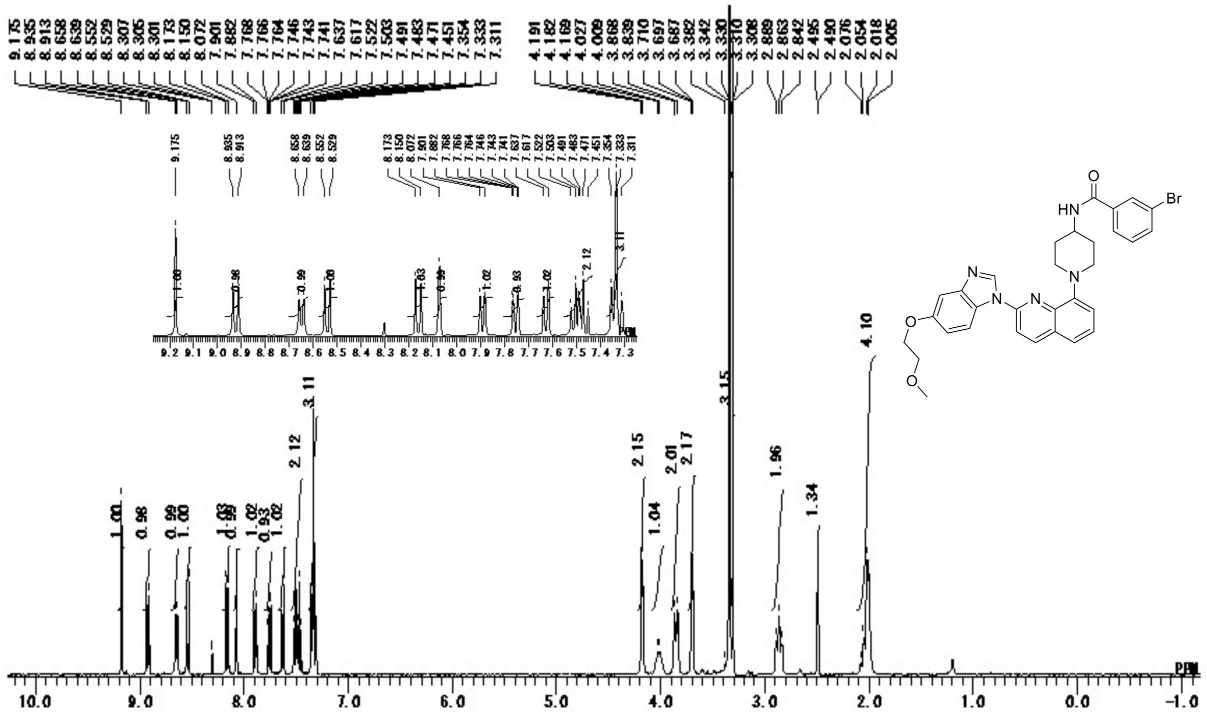

9. ^{13}C-NMR: **5** (DMSO-d_6)

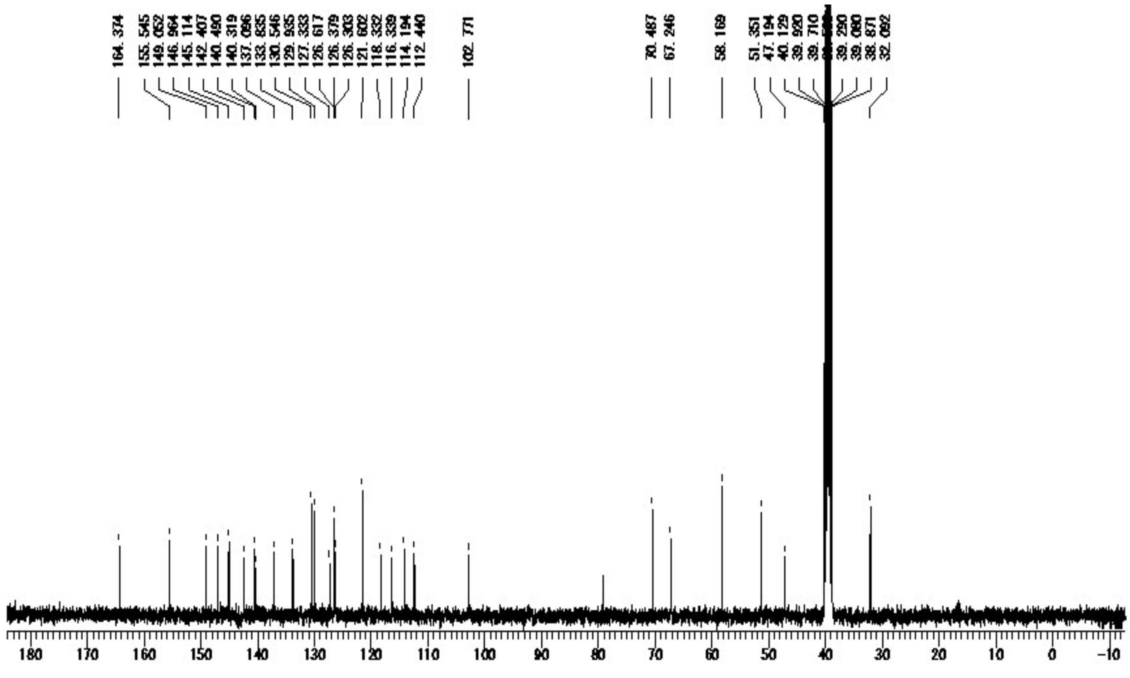

Supplement: Supplementary file 1 — Supplementary Information [file 41598_2018_28529_MOESM1_ESM.pdf]
